# Efficacy and Safety of Intralesional Purified Protein Derivative Versus Vitamin D for the Treatment of Cutaneous Warts: A Systematic Review and Meta-Analysis of Randomized Controlled Trials

**DOI:** 10.3390/jcm15103564

**Published:** 2026-05-07

**Authors:** Ahmed Abu-Zaid, Samira Hilali, Zainab Albasheer, Shouq Alkhatlan, Fanr F. Alraqum, Abdulmuhsin H. Al-Rashid, Malak A. Alshamali, Faisal Fahad AlSuwailem, Abdullah N. Almutairi, Waleed Alfailakawi, Abdullah M. Alharran

**Affiliations:** 1 College of Medicine, Alfaisal University, Riyadh 11533, Saudi Arabia; 2 Department of General Surgery, Sheikh Jaber Al-Ahmad Al-Sabah Hospital, Ministry of Health, Kuwait City 13018, Kuwait; 3 Kuwait Institute for Medical Specialization, Kuwait City 13018, Kuwait; 4 Faculty of Medicine, Alexandria University, Alexandria 21526, Egypt; 5 College of Medicine and Medical Sciences, Arabian Gulf University, Manama 329, Bahrain

**Keywords:** immunotherapy, PPD, HPV, dermatology, plantar

## Abstract

**Background**: Cutaneous warts, caused by HPV, are common, and conventional destructive treatments often fail to prevent recurrence. Intralesional immunotherapy offers a promising alternative. Specifically, purified protein derivative (PPD) and vitamin D have demonstrated efficacy; however, a direct comparison of their therapeutic value and safety profiles is needed. This systematic review and meta-analysis of randomized controlled trials (RCTs) aimed to compare the efficacy and safety of intralesional PPD versus intralesional Vitamin D for cutaneous warts. **Methods**: A comprehensive search of PubMed, Scopus, CENTRAL, and Google Scholar was conducted for RCTs up to November 2025. Primary outcomes were the complete and partial clinical response rates of warts. Secondary outcomes included recurrence and adverse events. Risk ratios (RRs) with 95% confidence intervals (CIs) and *p*-values were pooled using STATA 19.5. **Results**: Seven RCTs involving 520 patients were included. The analysis found no significant difference in the rate of complete clinical response (RR: 1.07, 95% CI [0.95, 1.21]; *p* = 0.24) or partial clinical response (RR: 0.90, 95% CI [0.56, 1.46]; *p* = 0.68) between the two groups. Also, the two groups showed no significant difference in recurrence rate (RR: 1.14, 95% CI [0.48, 2.68]; *p* = 0.77). Regarding safety, PPD was associated with a significantly higher risk of injection site erythema (RR: 4.76, 95% CI [2.08, 10.93]; *p* < 0.001) and flu-like symptoms/fever (RR: 8.28, 95% CI [1.59, 43.18]; *p* = 0.01). Still, no significant differences were found for injection site pain (*p* = 0.12) or swelling (*p* = 0.68). **Conclusions**: With uncertain evidence, intralesional PPD and vitamin D demonstrate comparable overall efficacy in clearing cutaneous warts. However, PPD carries a higher risk of systemic adverse events (fever) and injection site erythema, with a more effective potential to clear distant warts. The choice between the two agents should be based on the clinical profile and patient preference.

## 1. Introduction

Verrucae, also known as cutaneous warts, are widespread, benign epidermal proliferations caused by human papillomavirus (HPV) [1]. Cutaneous warts have a high prevalence, representing a significant burden, as they can cause cosmetic disfigurement, pain, and social stigma [2]. The management of cutaneous warts is often challenging, as they are known for their high recurrence rates and resistance to therapy [3]. This challenge is particularly noticeable in cases involving multiple, recalcitrant, or palmoplantar lesions [4].

Standard first-line treatments are primarily locally destructive, including topical salicylic acid, cryotherapy, and laser ablation [3]. Still, these methods present significant limitations, as they are often painful, carry risks of scarring or dyspigmentation, and are impractical for patients with multiple lesions [5,6]. Additionally, these methods may fail to clear the HPV from adjacent tissues, resulting in high recurrence rates [7]. These limitations highlight a significant therapeutic gap, underscoring the need for treatments that are not only effective but also target the viral cause, are well-tolerated, and can prevent future recurrence.

Intralesional immunotherapy has emerged as a promising alternative that addresses this gap [8]. Its core mechanism involves injecting an antigen to stimulate the host’s cell-mediated immunity to recognize and eradicate HPV-infected cells [7,9]. A key advantage of this approach is its ability to induce a systemic immune response, often leading to the resolution of both the injected wart and distant, untreated lesions [10]. Purified protein derivative (PPD) and Vitamin D are among the various investigated agents. PPD is a well-established recall antigen that stimulates a non-specific Th1 immune response [11,12]. Importantly, the efficacy of intralesional PPD is dependent on pre-existing host sensitization to *Mycobacterium tuberculosis* antigens, as it induces a delayed-type hypersensitivity (Th1) recall response rather than a primary immune reaction; thus, therapeutic outcomes are greater in previously sensitized individuals (e.g., BCG-vaccinated), while a lack of sensitization may reduce response rates [13]. Vitamin D is a more recently investigated agent with multiple immunomodulatory properties, including the regulation of epidermal cell proliferation and cytokine production [14,15].

More recently, several head-to-head randomized controlled trials (RCTs) have been conducted to compare intralesional PPD and Vitamin D_3_ directly [16,17,18,19,20,21,22]. However, these individual studies often have small sample sizes and have reported conflicting results. Therefore, the objective of this study was to systematically synthesize all available RCTs to compare the efficacy and safety of intralesional PPD versus Vitamin D for the treatment of cutaneous warts, informing the clinical practice of the most optimal immunotherapeutic approach.

## 2. Methods

### 2.1. Protocol Registration

The systematic review was registered in PROSPERO with the following CRD: CRD420261324818. This research was conducted in alignment with the PRISMA guidelines [23] and the Cochrane Handbook for Systematic Reviews of Interventions [24]. The PRISMA checklist is provided in Table S1.

### 2.2. Sources of Data and Search Approach

On 10 November 2025, A.A. and S.H. conducted a systematic literature search across several electronic databases, including the Cochrane Central Register of Controlled Trials (CENTRAL), Scopus, PubMed, and Google Scholar. The search strategy utilized the following search string: (PPD OR tuberculin OR “purified protein derivative”) AND (“vitamin D” OR “vitamin D_3_” OR cholecalciferol) AND (wart* OR verruca OR verrucae OR condyloma OR “human papillomavirus” OR hpv). A comprehensive summary of search queries and database results is provided in Table S2. In addition, the reference sections of all included trials were reviewed manually to ensure comprehensive coverage and prevent the omission of any relevant studies.

### 2.3. Eligibility Criteria

RCTs were included if they followed the following Population, Intervention, Control, and Outcome (PICO) framework:

- Population (P): patients with cutaneous warts, regardless of their type and location.
- Intervention (I): intralesional PPD, regardless of the dosing regimen.
- Control (C): intralesional vitamin D, regardless of the dosing regimen.
- Outcomes (O): the primary outcomes were clinical response (complete response (100% disappearance of the wart), partial response of the injected warts (>50% to 99% reduction)). Secondary outcomes included complete/partial responses of the distant warts, recurrence rate, and adverse events.

The exclusion criteria encompassed single-arm clinical studies, quasi-experimental studies, conference abstracts or proceedings, study protocols, and observational studies or reviews.

### 2.4. Study Selection

Two investigators independently assessed the eligibility of all identified records using Covidence software (https://www.covidence.org/, accessed date: 11 November 2025). After automatic removal of duplicate entries, the remaining unique studies were screened in two phases. First, titles and abstracts were evaluated, followed by a full-text review of all potentially relevant articles. Any discrepancies between the investigators were resolved through discussion.

### 2.5. Data Collection

Data were independently extracted by two investigators, and any disagreements were resolved through discussion or, when required, by consulting the senior investigator. A structured Microsoft Excel sheet was created for this purpose and was pilot-tested prior to full data extraction. The extraction tool was divided into three key domains:

- Study-level data: including study ID, country of origin, study design, total number of participants, intervention details, treatment frequency, wart type, main inclusion criteria, primary outcomes, and follow-up period.
- Participant characteristics at baseline: such as age and gender, disease duration, family history, number of warts, size of warts, location of warts, and recalcitrance.
- Outcome data include clinical response, recurrence rate, and adverse events.

### 2.6. Quality Assessment and Certainty of Evidence

The quality of the studies and the risk of bias in each RCT were assessed using the revised Cochrane Risk of Bias tool (RoB 2) [25]. Two investigators independently evaluated the studies as per protocol. Any differences in their assessments were resolved through discussion and agreement. The overall strength of the evidence was also evaluated using the GRADE approach [26,27]. All judgments were recorded as per protocol by two investigators, and any disagreements were resolved through discussion.

### 2.7. Data Synthesis

The statistical analyses were performed using Stata/SE version 19.5 (StataCorp LLC, College Station, TX, USA). The risk ratio (RR) was calculated for dichotomous outcomes, presented alongside its corresponding 95% confidence intervals (CIs). A fixed-effect model was used for the main analysis. However, when significant heterogeneity was found, a random-effects model using REML was applied. Heterogeneity was assessed using the chi-square (χ^2^) test and the I^2^ statistic, with a *p*-value < 0.1 or an I^2^ value ≥ 50% indicating significant heterogeneity. When heterogeneity was present, a leave-one-out sensitivity analysis was conducted to test the robustness of the results. Publication bias could not be assessed because all outcomes included fewer than 10 randomized controlled trials [28].

## 3. Results

### 3.1. Summary of Literature Identified

**Figure 1:**
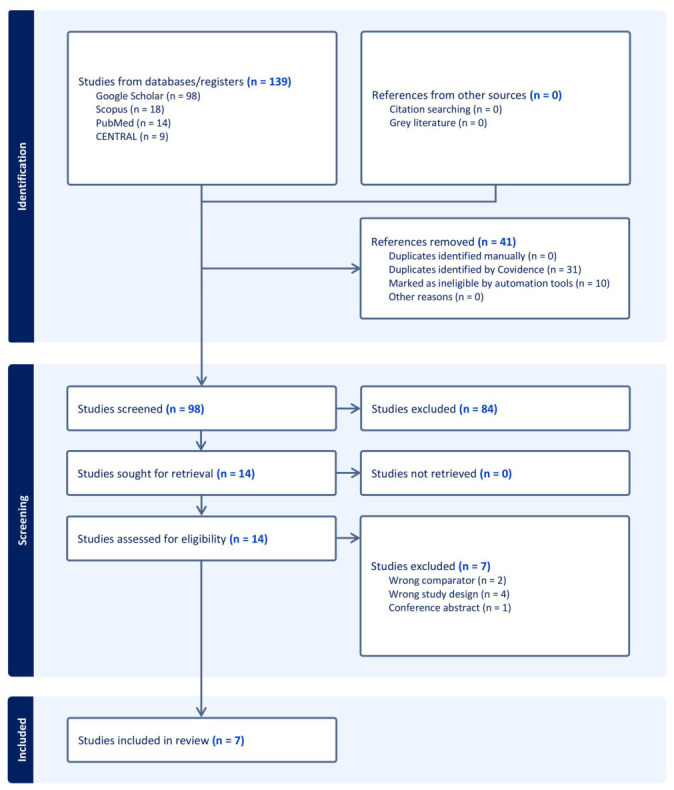
PRISMA flowchart of the screening process.

Collectively, 139 citations were charted. After deleting 41 duplicates, 98 records were screened, and 84 were excluded. Fourteen full-text articles were assessed, with 7 excluded for various reasons (Table S2). Finally, seven studies [16,17,18,19,20,21,22] were analyzed in this research (Figure 1).

### 3.2. Summary of Analyzed RCTs

We analyzed seven RCTs and 520 patients [16,17,18,19,20,21,22]. The majority were carried out in India (five trials), with two studies from Egypt. The included patients ranged from children to adults, presenting with various types of cutaneous warts. Treatment typically involved injecting the largest wart every 2–4 weeks for a maximum of 3–4 sessions, though specific dosages differed across trials. Additional information on the design of the included studies is provided in Table 1. Baseline characteristics of the included patients are presented in Table 2.

### 3.3. Summary of Quality Assessment and Certainty of Evidence

**Figure 2:**
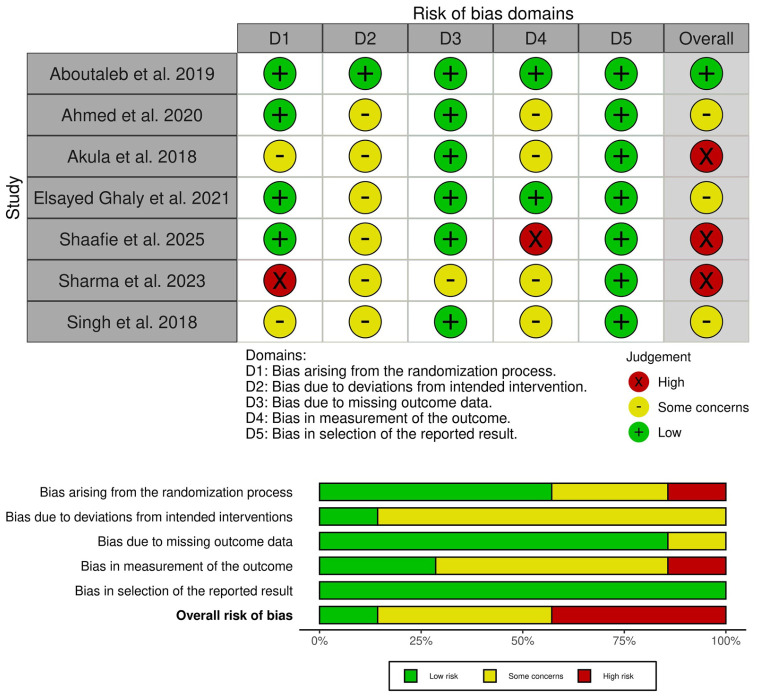
Quality assessment of risk of bias in the included trials. (**upper**) panel presents a schematic representation of risks (low = green, unclear = yellow, and high = red) for specific types of biases of the studies in the review. (**lower**) panel presents risks (low = red, unclear = yellow, and high = red) for the subtypes of biases of the combination of studies included in this review [16,17,18,19,20,21,22].

One trial exhibited an overall low risk of bias [20], three showed some concerns [16,18,22], and three exhibited a high risk of bias [17,19,21]. For selection bias, Akula et al. showed some concerns owing to the absence of clear information on the randomization method, and Sharma et al. exhibited a high risk of bias, as they used an open list of simple random tables [17]. Most trials raised concerns about performance and detection biases owing to the open-label design or lack of information on blinding. Finally, Sharma et al. exhibited issues relating to attrition bias, as 13% of patients were lost (15/115) without clear reasons [17]. Summary of the quality assessment of the included studies is summarized in Figure 2. The details of the certainty of the evidence assessment are presented in Table 3.

### 3.4. Primary Outcomes: Response Rates

**Figure 3:**
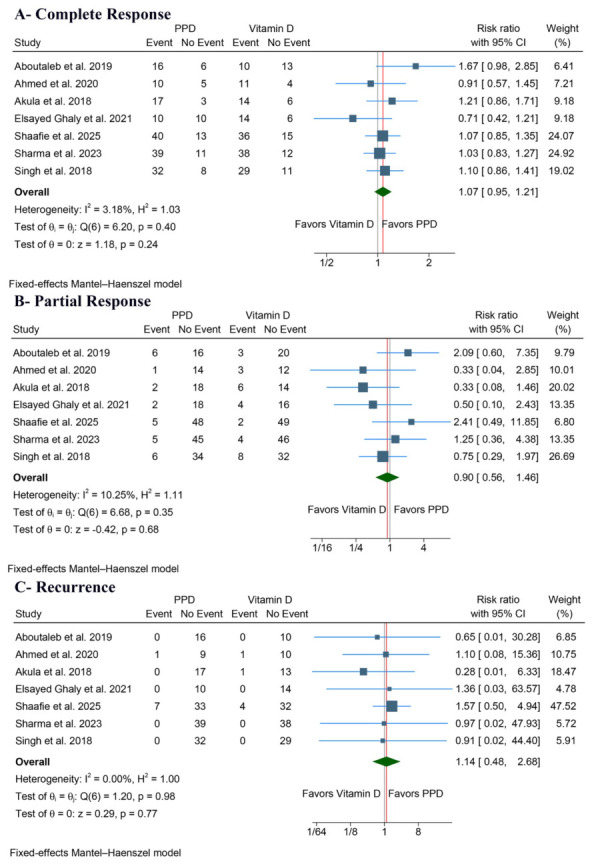
Forest plots of the efficacy outcomes, CI: confidence interval [16,17,18,19,20,21,22].

No significant difference was observed in the rate of complete clinical response between PPD and vitamin D (RR: 1.07, 95% CI [0.95, 1.21], *p* = 0.24) (Figure 3A). Pooled studies showed low heterogeneity (I^2^ = 3.18%). Additionally, there was no significant difference in the rate of partial clinical response between the two groups (RR: 0.90, 95% CI [0.56, 1.46], *p* = 0.68) (Figure 3B). Pooled studies also showed low heterogeneity (I^2^ = 10.25%).

### 3.5. Secondary Outcomes

#### 3.5.1. Recurrence Rate

No significant difference was observed in the rate of recurrence between PPD and vitamin D (RR: 1.14, 95% CI [0.48, 2.68], *p* = 0.77) (Figure 3C), with no heterogeneity detected (I^2^ = 0.00%).

#### 3.5.2. Response of the Distant Warts

Two trials provided separate quantitative data for this outcome, both suggesting an advantage for PPD. Sharma et al. reported that PPD was statistically superior at clearing distant lesions; 70% (35/50) of the PPD group had a complete distant response, compared to only 48% (29/50) of the vitamin D group (*p* = 0.04) [17]. Similarly, Elsayed Ghaly et al. reported a complete distant response in 37.5% (3/8) of PPD patients, compared to 25% (1/4) of vitamin D patients [22]. This study also exhibited a significant link between the target wart’s response and the distant warts’ response in the PPD group (r = 0.775, *p* = 0.027) [21].

#### 3.5.3. Adverse Events

##### Injection Site Pain

**Figure 4:**
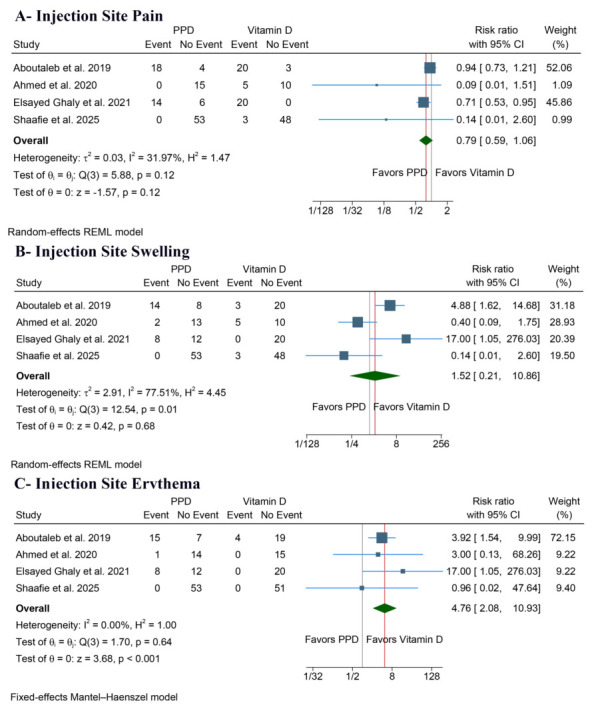
Forest plots of the safety outcomes, CI: confidence interval [16,19,20,22].

There was no significant difference between PPD and vitamin D (RR: 0.79, 95% CI [0.59, 1.06], *p* = 0.12, I^2^ = 31.97%) (Figure 4A). The sensitivity analysis showed that the results were stable (Figure S1) [16,19,20,22]. Also, the Galbraith plot identified the study by Elsayed Ghaly et al. as a potential outlier (Figure S2).

##### Injection Site Swelling

There was no significant difference between PPD and vitamin D (RR: 1.52, 95% CI [0.21, 10.86], *p* = 0.68), but with significant heterogeneity (I^2^ = 77.51%) (Figure 4B). The sensitivity analysis showed that the results were stable (Figure S3) [16,19,20,22]. Also, the Galbraith plot identified Aboutaleb et al. and Elsayed Ghaly et al. as sources of heterogeneity (Figure S4).

##### Injection Site Erythema

PPD significantly increased the risk compared to vitamin D (RR: 4.76, 95% CI [2.08, 10.93], *p* < 0.001), without evidence of heterogeneity (I^2^ = 0.00%) (Figure 4C).

##### Pruritus

**Figure 5:**
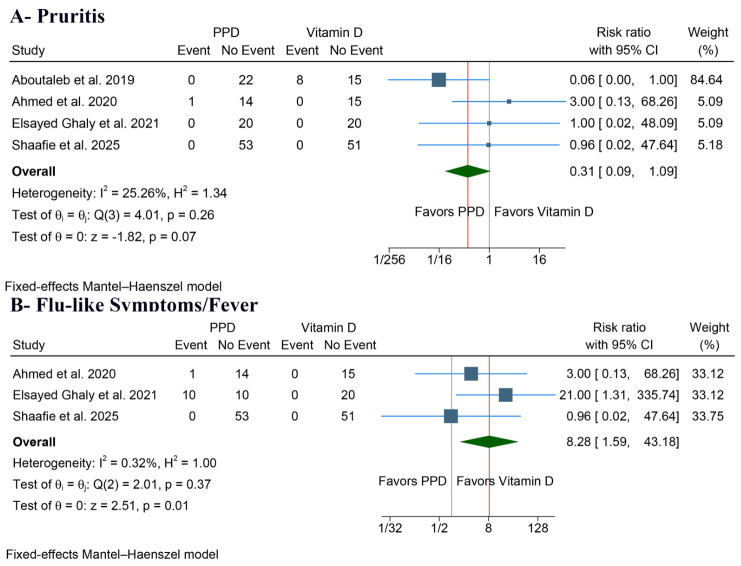
Forest plots of additional safety outcomes, CI: confidence interval [16,19,20,22].

There was no significant difference (RR: 0.31, 95% CI [0.09, 1.09, *p* = 0.07, I^2^ = 25.26%) (Figure 5A).

##### Flu-like Symptoms/Fever

PPD was significantly linked to a greater rate of flu-like symptoms or fever (RR: 8.28, 95% CI [1.59, 43.18], *p* = 0.01, I^2^ = 0.32%) (Figure 5B).

## 4. Discussion

Our synthesis of seven RCTs, involving 520 patients, showed that both agents are effective with comparable therapeutic outcomes, and there is no statistically significant difference between PPD and Vitamin D regarding complete and partial wart clearance. Also, our analysis showed that both intralesional PPD and vitamin D are associated with low recurrence rates, with no statistically significant difference observed between the two interventions. Finally, both treatments were generally well-tolerated, but PPD was associated with a significantly higher risk of injection site erythema and flu-like symptoms or fever.

The comparable efficacy of the two agents is achieved through different but overlapping immunological mechanisms. On the one hand, the efficacy of PPD may be dependent on the host’s pre-existing sensitization to *Mycobacterium tuberculosis* antigens, typically acquired through BCG vaccination or natural exposure [13,29]. When injected intralesionally, PPD stimulates a delayed-type hypersensitivity reaction, characterized by a robust Type IV immune response [30]. This reaction recruits non-specific inflammatory cells and stimulates the release of Th1 cytokines, including Interleukin-12 (IL-12) and Interferon-gamma (IFN-γ) [30].

The included trial by Aboutaleb et al. supported this hypothesis, demonstrating significant elevations in serum IL-12 and IFN-γ levels following PPD injection, which were not as pronounced with vitamin D [20]. These cytokines are crucial for attracting cytotoxic T cells and natural killer cells to the infection site, where they recognize and eliminate HPV-infected keratinocytes [31]. However, this systemic increase in pro-inflammatory cytokines also explains the notably higher incidence of flu-like symptoms and fever reported in our meta-analysis. Overall, intralesional immunotherapy should therefore be viewed primarily as an adjunctive therapeutic strategy rather than a definitive monotherapy for cutaneous warts, particularly in recalcitrant or multifocal disease.

On the other hand, the mechanism of vitamin D is more complex and multimodal. Unlike PPD, vitamin D does not depend on previous antigenic exposure. Alternatively, vitamin D influences immune responses by activating vitamin D receptors on keratinocytes and immune cells [15,32]. This activation leads to the upregulation of antimicrobial peptides, especially cathelicidin, which may contribute to antiviral defenses in epithelial tissues and could help inhibit HPV infection [33,34]. Additionally, vitamin D regulates epidermal cell differentiation, inhibiting keratinocyte proliferation, thus counteracting the hyperproliferative pathology of the wart [35]. The localized nature of this VDR-mediated response likely explains why vitamin D is associated with fewer systemic adverse events, such as fever. Still, it may cause local reactions, like pruritus, potentially due to the vehicle or localized histamine release [14].

Moreover, a significant benefit of intralesional immunotherapy, compared to ablative methods, is its capacity to eradicate distant warts [8,36]. Our qualitative synthesis supports this systemic immunomodulatory effect for both PPD and vitamin D. To clarify, Sharma et al. observed that PPD was statistically superior in clearing distant warts (70% vs. 48%, *p* = 0.04) [17]. At the same time, Elsayed Ghaly et al. also noted clearance of distant lesions in both groups, though with a non-significant trend favoring PPD (37.5% vs. 25%) [22]. This ability to induce clearance of distant warts is particularly beneficial in clinical practice, especially in patients with multiple or recalcitrant lesions, where individual lesion injection may be impractical or associated with significant discomfort. Importantly, despite the observed immunologic effects of intralesional agents, the management of distant warts remains a significant clinical challenge, and to date, no consistently effective treatment has been established to reliably target distant lesions. Vitamin D demonstrates only limited and inconsistent efficacy in this regard, reinforcing the need for systemic immunomodulatory approaches.

### Strengths & Limitations

This study represents the first systematic review and meta-analysis to compare the head-to-head efficacy of PPD versus vitamin D for wart treatment. By focusing exclusively on RCTs and excluding non-randomized studies, we provide the highest available level of evidence for this specific comparison. Also, the methodology strictly adhered to PRISMA guidelines, ensuring transparency and reproducibility.

However, our results remain constrained by the following: First, most included studies were assessed as having a high risk of bias or some concerns. Performance bias was the primary contributing factor, as the disparity in physical properties between the solutions (aqueous PPD and oily vitamin D) and the visible local responses (erythema with PPD) rendered double-blinding nearly impossible. Second, the primary outcome of wart clearance relies on clinical observation. In the absence of blinded outcome assessment in several trials, this introduces a risk of detection bias. Third, we observed significant statistical heterogeneity in some safety outcomes. Variations in injection techniques, administered volumes, and the definitions and reporting of adverse effects across various centers may explain this variability. Fourth, although we pooled data from seven trials, the total sample size of 574 patients remains relatively small. This limits the statistical power to detect rare adverse events or slight differences in efficacy subgroups, such as specific wart types. Fifth, an additional important limitation is the short follow-up duration in most included trials. This raises the possibility that what is reported as ‘recurrence’ may in fact represent incomplete initial clearance rather than true recurrence of infection, particularly given the well-known ability of HPV to persist subclinically in adjacent tissue. Finally, the previous limitations rendered our certainty assessment low or very low; therefore, our results should be interpreted with caution.

## 5. Conclusions

Intralesional PPD and vitamin D show broadly comparable efficacy in the clearance of cutaneous warts and in reducing recurrence rates. Therefore, the choice between these agents should not be guided by efficacy alone, but should also take into account safety profiles and patient preferences. PPD is associated with a stronger systemic immune response, which may increase the incidence of influenza-like symptoms and local erythema; however, this heightened immunologic activation may also contribute to the clearance of distant (non-injected) warts. In contrast, vitamin D demonstrates similar overall cure rates but is generally better tolerated, with a more favorable systemic side-effect profile. Despite these findings, the current evidence base remains heterogeneous, and definitive conclusions are limited by variability in study design, dosing regimens, and outcome measures. Future research should therefore prioritize large, well-designed, double-blinded RCTs using standardized protocols to better define comparative efficacy and safety. Overall, intralesional PPD and vitamin D are best considered adjunctive rather than stand-alone therapies. Their optimal use likely lies within a multimodal treatment approach tailored to individual patient characteristics, safety considerations, and clinical context.

## Figures and Tables

**Table 1:** Summary characteristics of the included RCTs.

Study ID	Study Design	Country	Total Participants	PPD Group Details	Vitamin D Group Details	Application Frequency	Treatment Duration	Wart Type	Main Inclusion Criteria	Primary Outcome	Follow-Up Duration
Aboutaleb et al. 2019 [20]	RCT	Egypt	45	0.1–0.3 mL (5 TU) based on skin test	0.6 mL (200,000 IU/1.5 mg)	Every 3 weeks	Maximum 3 sessions	Multiple extragenital (common and/or plantar)	Patients (>12 years) with multiple extragenital warts	Clinical efficacy (clearance) and safety	3 months
Ahmed et al. 2020 [16]	RCT	India	30	0.1 mL (10 TU)	2 units (600,000 IU/15 mg/mL)	Every 2 weeks	Minimum 3 sessions (up to 8)	Cutaneous (palmoplantar, plane, vulgaris, filiform)	Patients (10–70 years) with cutaneous warts	Efficacy (clinical clearance)	6 months
Akula et al. 2018 [21]	RCT	India	40	0.2 mL (5 TU/mL) per wart	0.2 mL (15 mg/mL/600,000 IU) per wart	Every 2 weeks	Until complete clearance (avg. 3–4 sessions for PPD, >6 for Vit D_3_)	Verruca vulgaris, plantar, and periungual	Patients (>18 years) with single or multiple viral warts	Efficacy (clinical response)	3 months
Elsayed Ghaly et al. 2021 [22]	RCT	Egypt	40	0.3 mL (5 TU/0.1 mL)	0.2 mL (300,000 IU/7.5 mg/mL)	PPD: Every 2 weeks. Vit D_3_: Every 4 weeks.	Maximum 3 sessions	Plantar warts	Patients (≥10 years) with plantar warts	Efficacy (clinical response) and safety	6 months
Shaafie et al. 2025 [19]	RCT	India	104	0.1 mL (5 TU)	0.2 mL (120,000 IU)	Every 2 weeks	Maximum 4 sessions	Recurrent/recalcitrant extra-genital cutaneous (all types)	Patients (18–70 years) with ≥2 recalcitrant extra-genital cutaneous warts	Clinical efficacy (response) and safety	6 months
Sharma et al. 2023 [17]	RCT	India	100	0.1–0.2 mL	0.1–0.2 mL (600,000 IU/15 mg/mL)	Every 4 weeks	Maximum 4 sessions	Recalcitrant extragenital warts	Patients (12–65 years) with ≥2 recalcitrant extragenital warts	Efficacy (clinical response) and safety	3 months
Singh et al. 2018 [18]	RCT	India	80	0.1 mL (10 TU)	0.5 mL (600,000 IU/15 mg/mL)	Every 2 weeks	Maximum 4 sessions	Viral warts (filiform, vulgaris, palmoplantar, periungual)	Patients with single or multiple viral warts	Safety and efficacy (clinical response)	3 months

avg.: Average; IU: International Units; mg: Milligram; mL: Milliliter; PPD: Purified Protein Derivative; RCT: Randomized Controlled Trial; TU: Tuberculin Units; Vit D_3_: Vitamin D_3_.

**Table 2:** Baseline characteristics of the participants.

Study ID	Number of Patients in Each Group		Age (Years), Mean (SD)		Gender (Male), N (%)		Disease Duration, Mean (SD)		Number of Warts, Mean (SD)		Location of Warts		Recalcitrance	
	PPD	Vitamin D	PPD	Vitamin D	PPD	Vitamin D	PPD	Vitamin D	PPD	Vitamin D	PPD	Vitamin D	PPD	Vitamin D
Aboutaleb et al. 2019 [20]	22	23	31.13 (6.86)	32.13 (13.34)	18 (81.8%)	13 (56.5%)	1.69 (1.59) Years	1.58 (3.13) Years	9.81 (6.57)	10.57 (8.72)	Plantar (54.5%), Common (18.2%), Both (27.3%)	Plantar (43.5%), Common (39.1%), Both (17.4%)	Not specified	Not specified
Ahmed et al. 2020 [16]	15	15	NR	NR	NR	NR	NR	NR	NR	NR	NR (Total: 57.8% Palmoplantar, 20% Plane, 17.8% Vulgaris, 4.4% Filiform)		No	No
Akula et al. 2018 [21]	20	20	26.95 (5.49)	25.1 (4.41)	11 (55%)	13 (65%)	NR	NR	NR	NR	Verruca Vulgaris [11], Plantar [6], Periungual [3]	Verruca Vulgaris [12], Plantar [7], Periungual [1]	Not specified	Not specified
Elsayed Ghaly et al. 2021 [22]	20	20	23.20 (6.98)	25.90 (5.40)	14 (70%)	12 (60%)	6.52 (4.14) Months	8.72 (6.78) Months	Single: 12 (60%), Multiple: 8 (40%)	Single: 16 (80%), Multiple: 4 (20%)	Plantar warts only		No	No
Shaafie et al. 2025 [19]	53	51	28.09 (11.96)	26.63 (9.46)	42 (79.2%)	29 (56.9%)	NR	NR	NR	NR	V. filiformis [3], V. palmaris [3], Periungual [4], V. plantaris [11], V. plana [17], V. vulgaris [15]	V. filiformis [2], V. palmaris [5], Periungual [5], V. plantaris [10], V. plana [13], V. vulgaris [16]	Yes	Yes
Sharma et al. 2023 [17]	50	50	29.7 (5.3)	30.2 (6.1)	31 (62%)	29 (58%)	8.7 (2.2) Months	8.9 (2.6) Months	5.9 (3.1)	6.1 (2.9)	Extragenital warts		Yes	Yes
Singh et al. 2018 [18]	40	40	Total mean: 25.98		Total M:F = 51:29		Total mean: 6.7 months		Total: 80% multiple		Total: Palm 21, Feet 22, Face 16, Limb 10, Trunk 6		Yes	Yes

N: Number; NR: Not Reported; PPD: Purified Protein Derivative; SD: Standard Deviation; V.: Verruca.

**Table 3:** GRADE evidence profile.

Certainty Assessment						
Participants (Studies) Follow-Up	Risk of Bias	Inconsistency	Indirectness	Imprecision	Publication Bias	Overall Certainty of Evidence
Complete Response						
439 (7 RCTs)	very serious ^a^	not serious	not serious	not serious	none	⨁⨁◯◯ Low ^a^
Partial Response						
439 (7 RCTs)	very serious ^a^	not serious	not serious	Serious ^b^	none	⨁◯◯◯ Very low ^a,b^
Recurrence						
321 (7 RCTs)	very serious ^a^	not serious	not serious	very serious ^b,c^	none	⨁◯◯◯ Very low ^a,b,c^
Injection Site Pain						
219 (4 RCTs)	very serious ^a^	Serious ^d^	not serious	Serious ^b^	none	⨁◯◯◯ Very low ^a,b,d^
Injection Site Swelling						
219 (4 RCTs)	very serious ^a^	very serious ^e^	not serious	very serious ^b,c^	none	⨁◯◯◯ Very low ^a,b,c,e^
Injection Site Erythema						
219 (4 RCTs)	very serious ^a^	not serious	not serious	very serious ^b,c^	none	⨁◯◯◯ Very low ^a,b,c^
Pruritus						
219 (4 RCTs)	very serious ^a^	not serious	not serious	very serious ^b,c^	none	⨁◯◯◯ Very low ^a,b,c^
Flu-like Symptoms/Fever						
174 (3 RCTs)	very serious ^a^	not serious	not serious	extremely serious ^b,c^	none	⨁◯◯◯ Very low ^a,b,c^

Explanations: ^a^. Six out of seven trials showed serious concerns across several domains. ^b^. A wide confidence interval. ^c^. A low number of events. ^d^. I^2^ > 50%. ^e^. I^2^ > 75%.

## Data Availability

No new data were created or analyzed in this study.

## Supplementary Materials

The following supporting information can be downloaded at: https://www.mdpi.com/article/10.3390/jcm15103564/s1, Table S1: PRISMA 2020 checklist; Table S2: Search strategy; Table S3: Excluded records in full-text screening; Figure S1: Leave-one-out sensitivity analysis of injection site pain [16,19,20,22]; Figure S2: Galbraith plot of injection site pain; Figure S3: Leave-one-out sensitivity analysis of injection site swelling [16,19,20,22]; Figure S4: Galbraith plot of injection site swelling.
